# MALDI Mass Spectrometry Imaging: A Potential Game-Changer in a Modern Microbiology

**DOI:** 10.3390/cells11233900

**Published:** 2022-12-02

**Authors:** Maureen Feucherolles, Gilles Frache

**Affiliations:** Luxembourg Institute of Science and Technology (LIST), Advanced Characterization Platform, Materials Research and Technology, L-4422 Belvaux, Luxembourg

**Keywords:** MALDI MSI, Mass spectrometry imaging, microbiology, biomarkers, microbial interactions, diagnostics, drug distribution

## Abstract

Nowadays, matrix-assisted laser desorption/ionization time of flight mass spectrometry (MALDI-TOF MS) is routinely implemented as the reference method for the swift and straightforward identification of microorganisms. However, this method is not flawless and there is a need to upgrade the current methodology in order to free the routine lab from incubation time and shift from a culture-dependent to an even faster independent culture system. Over the last two decades, mass spectrometry imaging (MSI) gained tremendous popularity in life sciences, including microbiology, due to its ability to simultaneously detect biomolecules, as well as their spatial distribution, in complex samples. Through this literature review, we summarize the latest applications of MALDI-MSI in microbiology. In addition, we discuss the challenges and avenues of exploration for applying MSI to solve current MALDI-TOF MS limits in routine and research laboratories.

## 1. Modern Microbiology and Current Limitations

In all fields of microbiology, i.e., food safety, surveillance, infectiology or diagnostics, the key is to obtain accurate, swift and cost-efficient identification of microorganisms, as well as their different characteristics, such as antimicrobial resistance profiles or subtyping characteristics. On the one hand, culture-based phenotypic methods are widespread and are reference methods for several tests (e.g., antibiograms) due to their low costs. On the other hand, the up-to-date development and implementation of molecular technologies in routine microbiology is slowly replacing it [1]. Among these technologies, genomics and proteomics could be depicted as the two most used methods in microbiology laboratories.

On the genomics side, the high-discriminatory, next-generation sequencing could be used for a number of applications, ranging from species identification to genotyping for epidemiological investigation [2,3]. Therefore, sequencing could be easily compared as the Swiss-knife of the microbiologist. Nevertheless, its implementation in routine laboratories remains challenging due to wet (e.g., PCR amplification bias and sequencing errors) and dry (e.g., bioinformatic pipelines and data management) lab issues, as well as its overall costs [4]. On the proteomics side, the high-throughput matrix-assisted laser desorption/ionization-time of flight mass spectrometry (MALDI-TOF MS) has become the reference method in routine laboratories for the rapid and reliable identification of whole-cell microorganisms based on protein fingerprints [5]. Despite the initial price of the MALDI-TOF apparatus, i.e., approximately 180,000–200,000 euros, analysis of 96 samples only entail around 0.50 euros of chemicals and consumables [6]. Additionally, a maximum identification turnaround time of 25 min is required for 96 reliable identifications. Commercial MALDI-TOF MS systems include databases for a large panel of microorganisms—including bacteria [7], mycobacteria [8] and fungi [9]—of medical and food interest. Furthermore, many reports underlined the successful application of MALDI-TOF MS for identification of additional microorganisms—such as viruses [10,11,12], ectoparasites[13,14], protozoa and helminths [15,16,17]—as well as antimicrobial resistance profiles and subtyping in a research context [18,19]. In the post-genomics world, microbial proteomics will be the foremost complement to other omics-powered technologies as protein activity is the most important factor for understanding biological pathways.

Nevertheless, this methodology is not without flaws. Indeed, microbiologists frequently highlighted that commercial MALDI-TOF MS systems are struggling to identify closely related species (e.g., *Mycobacterium tuberculosis* complex and *Enterobacter cloacae* complex) based on their protein fingerprints [20]. For example, Saleeb and colleagues underlined that despite the fact that MALDI-TOF MS accurately classified all isolates as members of the *M. tuberculosis* complex, it was not possible to distinguish them into separate species [21]. The same observation was also highlighted by current research and for other mycobacterial complexes [22,23]. However, it is worth acknowledging the latest developments based on species-specific lipids to overcome such limitations, which was detailed elsewhere [24]. Along the same line, current MALDI-TOF MS microbial identification relies on either pure microbial monoculture streaked on agar plates or resuspended cells obtained by in-house or commercial protocols (e.g., Sepsityper^®^ kit) from complex biological samples (e.g., stools, urine and blood). At a time when there is a constant search for higher throughput, such an approach requires incubation time, at best 24 h and at worst up to 4 weeks, to allow microbial colonies to grow, which is not straightforward. Furthermore, although studies have shown that it may be possible to identify bi- or ternary bacterial mixtures without a purification step [25,26,27], it is currently not possible to identify a mixture of microorganisms, either in liquid samples or directly from biological samples, such as stools, using commercial settings. Moreover, the description of biomarkers is a panacea when it comes to distinguishing closely related species or specific antimicrobial resistance. Nevertheless, the MALDI-TOF MS apparatus used in routine laboratories does not allow *de novo* peptide sequencing in terms of resolution and ability to perform peptide fragmentation and, hence, identify those specific protein peaks. Biomarkers characterization is an important step if it is destined for clinics or food applications. For example, the biomarker identified by Griffin and colleagues, which was supposed to distinguish vancomycin-resistant and vancomycin-susceptible *Enterococcus faecium,* was not suitable for routine diagnostics [28]. Indeed, after peptides sequencing, the identified gene could not be directly linked to the presence of a *VanB* resistance gene [29]. According to introduced limitations related to the application of MALDI-TOF MS in microbiology, there is a need to upgrade the current methodology to free routine labs from incubation time and shift from a culture-dependent to an even faster culture-independent system.

Over the last years, numerous reviews focusing on the application of MALDI-TOF MS in microbiology have mentioned MALDI mass spectrometry imaging (MSI) as a potential new game-changer in both microbiology research and even diagnostics [30,31,32]. Along the same line, an increasing number of specialized reviews and books have described MSI technologies as an attractive tool for the future regarding the analysis of complex samples and microbiomes [33,34,35,36,37]. For example, Zou and colleagues published a review on MSI and its potential application in food microbiology to raise awareness of this technique [38]. On the one hand, MALDI-TOF MS is currently the reference method for the swift identification of microorganisms in routine laboratories [39]. On the other hand, Palmer and colleagues underlined in the results of their online survey that MALDI was the most popular ionization technique among respondents, with 95% of labs using this technology [40]. Therefore, the present paper will be focusing on MSI based on MALDI ionisation. The aim of this review is to summarize the current literature covering the application of MALDI-MSI in all microbiological fields. Throughout this paper, avenues of exploration for applying MSI to solve current MALDI-TOF MS limits in routine and research laboratories will be discussed.

## 2. Mass Spectrometry Imaging: A Picture Is Worth a Thousand Words

**Figure 1:**
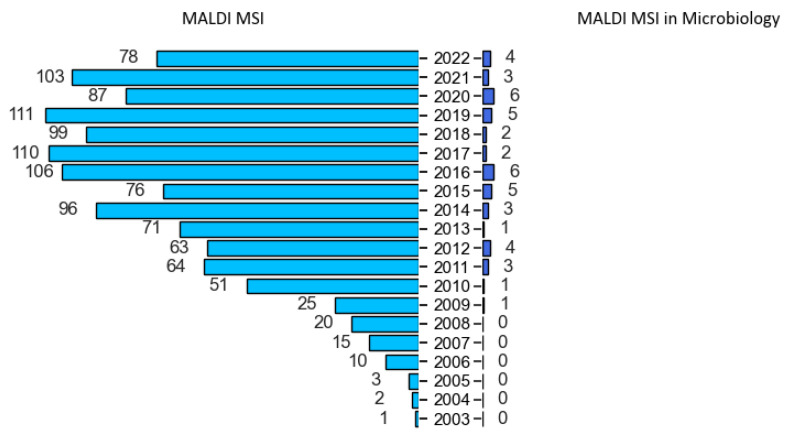
MALDI mass spectrometry imaging-related publications with the keywords “MALDI-MSI” or “MALDI mass spectrometry imaging” or “MALDI imaging” in Abstract or Title on PubMed search builder (searched in September 2022) compared to microbiology-related MALDI-MSI publications.

Over the last two decades, MALDI-MSI gained a fast-growing popularity (Figure 1) due to its non-specific nature in detecting biomolecules, such as small metabolites, lipids, peptides and proteins in complex samples [41]. In contrast to targeted imaging, like immunohistochemistry, it is a powerful tool that can simultaneously investigate both chemical composition and the spatial distribution of different molecular species within the sample, giving insights into biological systems [42].

Several reviews extensively described the basic principles of such methods [42,43,44]. In life sciences, samples can be a variety of tissue sections (e.g., brain, liver or skin cross-sections, 5–20 µm in thickness), smears and even microbial colonies. First, samples are mounted and fixed or directly grown on a support, such as Indium Tin Oxide-coated glass slides (ITO) or MALDI targets. Second, samples are coated with a chemical so-called matrix, which enabled the extraction and desorption/ionization of biomolecules from the co-crystallized samples. As a classical MALDI approach, the matrix absorbs laser energy, and analytes are desorbed and ionized into the gas phase. Different matrices could be selected, depending on the analyte class investigated. For example, α-cyano-4-hydroxycinnamic acid (CHCA) and 2,5-dihydroxybenzoic acid (DHB) were highlighted for their “universal analysis” for metabolites and peptides in a positive ion-mode [43]. Interestingly, due to the mass range limitation of certain analyzers (e.g., Orbitrap, Q-TOFs), high-resolution MS of large proteins was challenging [43]. Nevertheless, by implementing an extra enzymatic digestion step, performed directly on-tissue, i.e., on-tissue digestion, fragments of large proteins, such as myelin basic proteins, were observed [45]. Then, the tissue is directly scanned either in a continuous raster mode, i.e., the sample is moved at a constant speed while the laser is shooting, or in a pixel-by-pixel mode, i.e., a fixed number of laser pulses is applied per pixel to record a single mass spectrum. Nowadays, the lateral resolution or pixel size obtained with current commercial instrument is approximately 10 µm, and 30 to 50 pixel per second could be acquire at a moderate mass resolution [41]. MALDI MS images are reconstructed based on the intensities of a given ion on a (*x, y*) grid over the surface of the sample. The final image creates a visualisation of the sample based on the mass-to-charge of molecular ions of interest measured directly from the sample [42].

MALDI-MSI combines numerous advantages, including high sensitivity, high throughput and molecular specificity [42]. While imaging results are very close to immunohistochemistry, MALDI-MSI has the advantage of investigating multiple molecules within a single run without labelling or modifying the native sample [42,44]. Such information is important when considering potential changes to the chemical, physical or biological functions of biomarkers by the tagging reagent [42]. Hence, the morphological and molecular integrity of the scanned tissue is maintained [46]. Additionally, MALDI-MSI is suitable for the analysis of biological samples as (1) it can deal with a wide range of molecular weights (ca.100 Da to 100 kDa), (2) it produces singly charged ions and (3) the laser can interrogate specific histological spatial areas [42,47].

All these last points have enabled the development of biomedical and pharmaceutical applications requiring spatial molecular analysis. One of the major applications of MALDI-MSI is the possibility to perform a mapping of the molecular distribution in classical biological research to further understand biological pathways. When applied in clinically relevant areas, imaging can provide better diagnoses and prognoses and assess treatment of the disease [41]. Several studies investigated MALDI-MSI for the screening of diseased tissues in oncology, neurology or endocrinology [48,49,50,51]. In the case of neurosciences, MALDI-MSI was applied for the investigation of neurodegenerative (e.g., Alzheimer’s or Parkinson’s disease) and psychiatric disorders (e.g., schizophrenia) at the molecular level [52]. Matsumoto and colleagues discovered an abnormal distribution of phosphatidylcholine lipid species in the cortical layer of the frontal cortex region after analysis of the post-mortem brain of a patient with schizophrenia [53]. Although it is difficult to draw conclusions from their feasibility study, it highlights the importance of linking biochemical mapping to brain function disorders. Furthermore, when combined with machine learning, it enabled the development of classifiers for the potential diagnosis of cancer. For example, Mittal and colleagues developed a 98% accurate supervised machine learning algorithm, based on MALDI imaging mass spectra, to distinguish colorectal tumours from healthy tissue [54]. Along the same line, imaging was also employed for pharmacokinetic studies, i.e., how drugs reach their site of action, to visualize the distribution of an administrated drug in tissue sections by detecting specific mass signals of the studied drug [55]. Imaging of drug localisation could be either qualitative or quantitative. For example, by micro-spotting calibration standard solutions at different concentrations onto tissues, tofacitinib molecules were quantified in human epidermis [56]. However, quantitative MSI is still in its beginning and further work should be undertaken to establish a consensus on how to generate calibrated curves to assess drugs’ concentration directly in tissues [57]. Nevertheless, MALDI-MSI is widely used for a comprehensive analysis of biomolecules, and such an approach was also applied to microbiology in the early 2000s.

## 3. Where Are We with MALDI Mass Spectrometry Imaging in Microbiology?

MALDI-TOF MS technology was introduced into diagnostics labs two decades ago. However, such an apparatus was already implemented long before in chemistry and biochemistry labs for biomolecules analysis [58]. In 2016, an online survey was conducted among the MSI community to determine user profiles, as well as their related applications [40]. Not surprisingly, the great majority of users were chemists, biochemists and biologists, and 80% of them applied MSI for the study of either small molecules, lipidomics, metabolomics or pharmaceutical studies. Microbiologists were the less represented profiles in this survey, and few applications revolved around microbiology applications. Nevertheless, Dorrestein’s group was the first lab to investigate MALDI-MSI applied to microbiology and diverse related topics (e.g., metabolic exchanges and profiling) [59,60,61,62,63,64,65,66,67,68,69,70,71,72]. Accordingly, they opened a world of possibilities to explore and better understand the microscopic world. Since then, several MALDI-MSI articles focusing on numerous microorganisms, such as bacteria, fungi, parasites, viruses and protozoa (Table 1 and Figure 1), and in many fields, such as clinical, food and environment, were published.

### 3.1. Microbial and Host-Microbes’ Interactions

**Figure 2:**
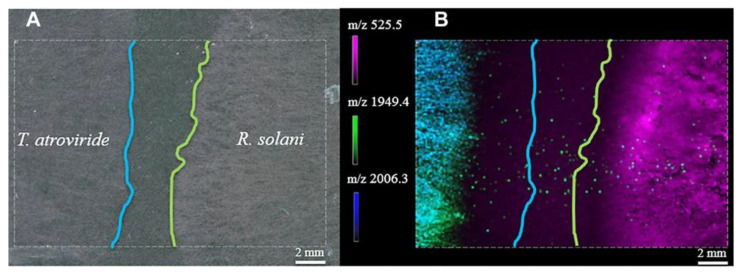
Light microscopic (**A**) and MALDI MSI (**B**) analysis of physically non-interacting *Trichoderma atroviride* and *Rhizoctonia solani* hyphae. Figure was reproduced with permission from Holzlechner and colleagues [74].

Current applications chiefly rely either on the interactions between two microorganisms, microbes and their hosts, or direct environment. In 2009, Yang and colleagues published the first ever and groundbreaking study underlining the possibility to do MSI of intact bacterial colonies grown on MALDI targets to study bacterial interactions. By using such an approach, they investigated interspecies interactions between *B. subtilis* and *S. coelicolor* involving several metabolites [60]. Since then, similar microbial interaction studies were achieved [62,63,65,66,68,73,74,75,76,77,78,79,80] (Figure 2). One of the latest was the investigation of the post-ionization (PI), also called MALDI-2, for imaging bacterial colonies [81]. The classical single laser MALDI approach produces low ions yield, so-called “lucky survivors”, resulting in mass spectra with abundant or easily ionizable analyte molecules [82,83]. Hence, low abundant or hardly ionizable molecules, which might play a key role in metabolism pathways, might be missed. One way to enhance ion yield without extra preparation is to use PI, where a second MALDI-like ionization event occurs, interacting with the already desorbed particles’ plume. As such, Brockmann and colleagues used this technique, to study *P. aeruginosa*, *S. aureus* and *B. subtilis* grown on polyamide membranes. In addition, the authors also produced MSI for the inhibition of *P. aeruginosa* when exposed to a β-lactam antibiotic disk. Overlay images revealed a 2 mm width structure where a high abundance of several 2-alkyl-quinolones, an important part of the quorum-sensing machinery of *P. aeruginosa*, was detected close to the inhibition zone.

**Table 1:** Specific microbial literature operating with MALDI mass spectrometry imaging. TOF: Time-of-Flight, LTQ: Linear Ion Trap, FT-ICR: Fourier-transform ion cyclotron resonance, Q: Quadrupole, NA: Not applicable.

Class	Main Objective	Organisms	Analyzer	Lateral Resolution	Year	References
**Bacteria**	Biofilm formation	*Bacillus*	TOF	NA	2015	[72]
			TOF/TOF	250 µm	2016	[84]
		*Pseudomonas*	TOF/TOF	100 µm	2014	[85]
		*Pseudomonas* *Staphylococcus*	TOF	50 µm	2016	[86]
		*Listeria*	TOF	100 µm	2018	[87]
	Biomarker identification	*Mycobacterium*	LTQ-orbitrap	50 µm	2018	[88]
	Drug effect	*Pseudomonas*	TOF	500 µm	2015	[70]
	Host-microbes’ interactions	Intracellular microbial communities of *Bathymodiolus*	Q-orbitrap	3 µm	2020	[89]
		*Pseudonocardia*	LTQ-orbitrap	75 µm	2017	[90]
		Gut microbiota	NA	50 µm	2022	[91]
		Gut microbiota	TOF/TOF	NA	2012	[67]
		*Streptomyces*	LTQ-orbitrap	NA	2011	[92]
		*Francisella*	FT-ICR	75 µm	2017	[93]
		*Escherichia* *Pseudomonas*	TOF/TOF	NA	2020	[94]
	Microbial interactions	*Pseudomonas* *Escherichia* *Staphylococcus*	Q-orbitrap	50 µm	2019	[76]
		*Bacillus* *Streptomyces*	TOF/TOF	NA	2012	[80]
		*Lysobacter* *Bacillus* *Pseudomonas* *Streptomyces* *Staphylococcus* *Mycobacterium*	TOF	200–800 µm	2012	[64]
		*Lysobacter*	TOF/TOF	50 µm	2015	[61]
		*Bacillus* *Staphylococcus*	TOF	200–350 µm	2011	[66]
		*Paenibacillus* *Bacillus*	TOF/TOF	300 µm	2019	[63]
		*Bacillus*	TOF	NA	2010	[65]
		*Pseudomonas*	TOF	400 µm	2016	[62]
		*Pseudomonas* *Aspergillus*	TOF/TOF	400–600 µm	2012	[68]
		*Bacillus* *Streptomyces*	TOF/TOF	NA	2009	[60]
		*Paenibacillus*	FT-ICR	NA	2013	[73]
	Sample preparation	*Myxobacteria*	TOF/TOF	350 µm	2015	[71]
		*Bacillus*	TOF/TOF	200 µm	2016	[95]
	Spatial distribution	Microbial mat	FT-ICR	25 µm	2020	[96]
**Fungi**	Microbial interactions	*Trichoderma* *Rhizoctonia*	TOF/TOF	NA	2016	[74]
	Host-microbes’ interactions	*Aspergillus*	FT-ICR	50 µm	2020	[78]
		*Aspergillus*	TOF/TOF	500 µm	2019	[79]
	Sample preparation	*Aspergillus*	TOF/TOF	35 µm	2014	[97]
**Parasites** **& Vectors**	Chemical characterization	*Schistosoma*	LTQ	50 µm	2014	[98]
			Q-orbitrap	5 µm	2020	[99]
		*Anopheles*	Q-orbitrap	12 µm	2015	[100]
	Drug distribution	*Schistosoma*	Q-orbitrap	5–9 µm	2021	[101]
		*Fasciola*	Q-orbitrap	10 µm	2022	[102]
			Q-orbitrap	10 µm	2020	[103]
	Host-microbes’ interactions	*Schistosoma*	Q-orbitrap	10 µm	2022	[104]
		Parasitic nematodes	TOF & Q-orbitrap	25–8 µm	2021	[105]
**Protozoa**	Method improvement	*Paramecium*	NA	1.4 µm	2016	[106]
	Host-microbes’ interactions	*Leishmania*	FT-ICR	50 µm	2021	[107]
**Viruses**	Biomarker identification	MDV	TOF/TOF	NA	2019	[108]
		Parvovirus	TOF/TOF	50 µm	2022	[109]
		HPV	TOF	NA	2011	[110]
	Microbial interactions	EhV201	FT-ICR	100 µm	2019	[77]

Therefore, by boosting the ion yield of important biomolecules, PI could be a relevant additional tool for the accurate analysis of chemical communication in microbial communities.

**Figure 3:**
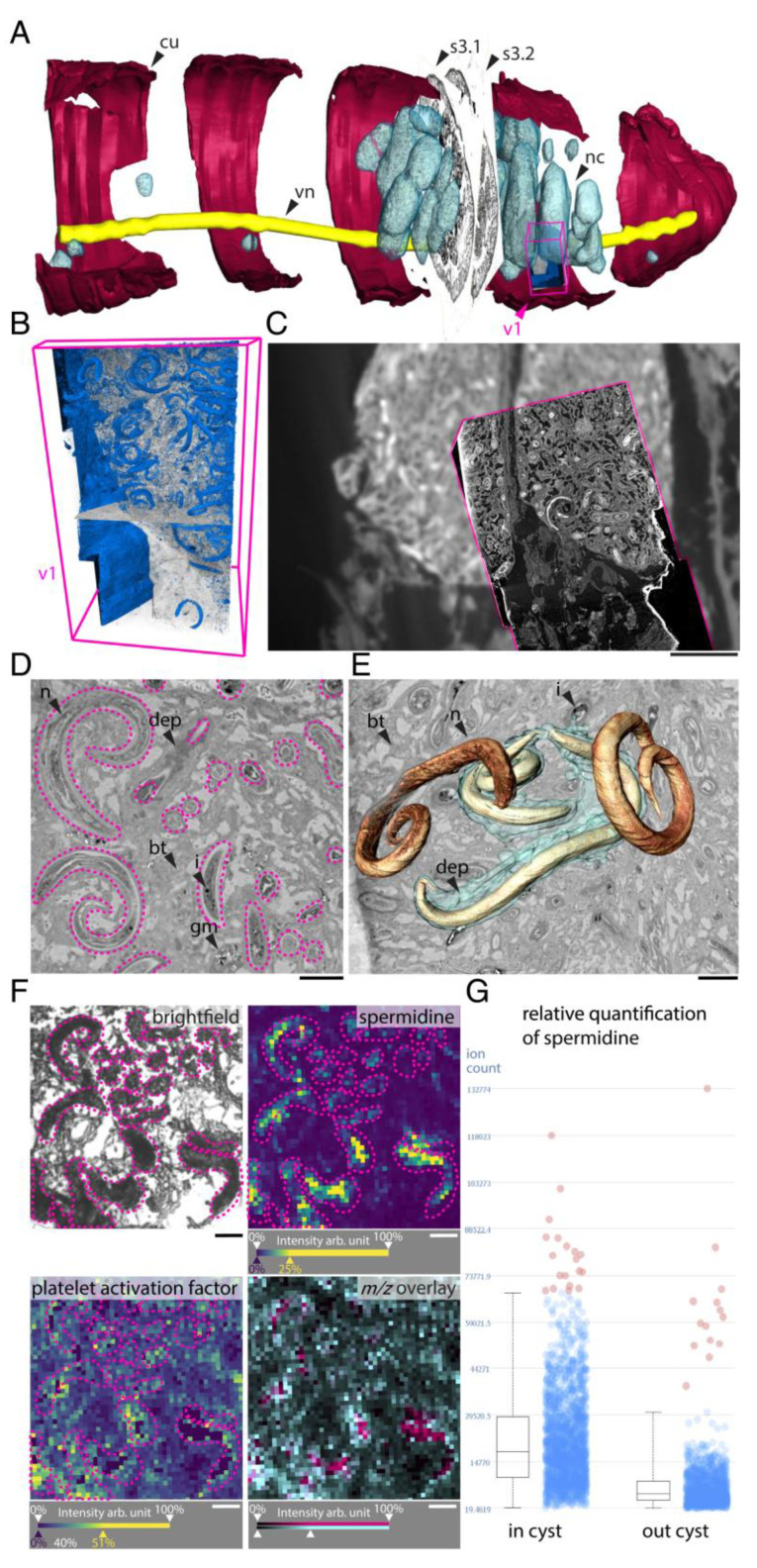
Using the 3D chemo-histo-tomography (CHEMIST) atlas to expose the interactions between adult earthworms (*L. rubellus*) and parasitic nematodes (*Rhabditis*). Figures were reproduced with permission from Geier and colleagues [105].

One of the biggest challenges of the 21st century is to further explore and understand metabolic interactions between symbiotic or pathogenic microorganisms and their host environment. Indeed, it was highlighted that unsuitable host-microbes interaction could trigger multiple chronic inflammatory diseases (e.g., inflammatory bowel disease or colorectal cancer) in humans [111]. In this sense, Geier and colleagues performed two studies investigating in situ spatial metabolomics in small animals, i.e., earthworms and mussels [89,105]. In the case of the earthworms, the authors introduced chemo-histo-tomography (CHEMHIST) combining MALDI-MSI and micro-computed tomography, allowing a three-dimensional (3D) map of its chemical and physical interactions with microorganisms, i.e., bacteria and parasitic nematodes, within the host (Figure 3) [105]. In the case of mussels as target organisms, Geier and colleagues presented imaging workflow combining fluorescence in situ hybridization (FISH) microscopy and high resolution MALDI-MSI (metaFISH), making it possible to visualize metabolic phenotypes and their associated partners [89]. Along the same line, Wiedemann and colleagues explored the interaction of parasitic *Schistosoma mansoni* trapped eggs (60–200 µm) in hamster liver. Biomarkers detected by liquid chromatography (LC) tandem mass spectrometry (MS/MS) and visualized by MALDI-MSI pointed out the lipid’s alteration after infection with *S. mansoni* and its eggs [104]. While MALDI-MSI was associated with other separation or complex imaging techniques, it was also reported to have been combined with scanning electron microscopy (SEM) for the visualization of *Aspergillus* and *Pseudomonas* in rat lungs [78].

In the end, regular or 3D MALDI-MSI alone or combined with other imaging modalities could be interesting for further explorations of host-microbes’ interactions, including human gut microbiota, symbiotic systems and other relevant pathogens such as *M. tuberculosis*.

### 3.2. Biofilms and Microbial Mats Formation

**Figure 4:**
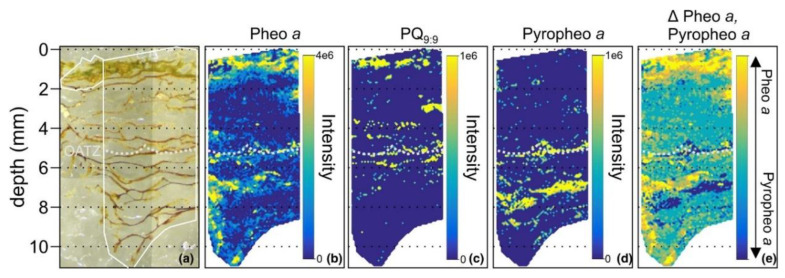
Images of the mat slice (**a**) and 75 µm resolution MSI of pheophytin (Pheo) *a*, plastoquinone (PQ9:9) and pyropheophytin *a* (**b**–**d**) depicting the spatial distribution of the oxygenic chlorophototroph *Synechococcus* spp. Panel (**e**) shows the difference between the normalized relative abundance of Pheo *a* and Pyropheophytin *a* (denoted as Δ Pheo *a*, Pyropheo *a*). Figure and caption were reproduced with permission from Wörmer and colleagues [96].

In some fields like microbial ecology, it is also crucial to identify the spatial distribution of microbial populations under specific structures, such as microbial mat, i.e., multi-layered sheets of microorganisms, or the closely related biofilm. In comparison to biofilm, microbial mat ranges from several millimeters to centimeters thickness, and it is stratified into distinct layers [112]. MALDI-MSI was applied for ecosystem inspection in order to better understand microbial population spatial distribution and, hence, functioning [96]. In the study of Wörmer and colleagues, an embedded microbial mat from a Yellowstone spring was investigated by MALDI-MSI to provide a description of the structure of the microbial mats. Based on the distribution of certain chloropigments, such as pheophytin, bacteriopheophytin and quinones, several chlorophototrophs groups, including the cyanobacteria *Synechococcus* spp. prominently in the upper 2 mm mat, were identified (Figure 4).

Along the same line, while some biofilms are innocuous, others may contribute to the etiopathogenesis of diseases, such as cystic fibrosis and urinary tract infection, or chronic infections [113]. Considering the large panel of diseases and infections directly linked to biofilms, there is a need to better understand biofilm structures. From this perspective, few studies considered MALDI-MSI to examine biofilm formation drivers, as well as bacterial interactions within biofilms [72,84,85,86,87]. *B. subtilis* is a popular bacterium model for studying biofilms due to the fact that it can develop different types of biofilms. In this context, Bleich and colleagues identified by MALDI-MSI that thiocillins, a *B. cereus*-produced peptide antibiotic, triggered biofilm formation in *B. subtilis*. Likewise, MSI combined with fluorescence was used to explore cellular and molecular heterogeneity in wild type and mutant *B. subtilis* biofilm [84].

Through the use of MALDI-MSI, complex microbial structures could be screened so as to deconstruct intricate molecular mechanisms.

### 3.3. Chemical Characterization and Visualization

**Figure 5:**
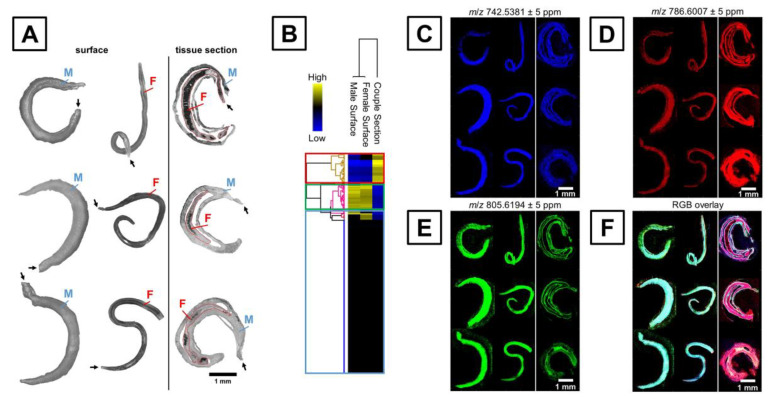
Light Microscopic and MALDI-MSI analysis of male (M) and female (F) *S. mansoni* tegument surface-associated lipids. Figure was reproduced from Kadesch and colleagues’ work [99].

Another important application of MALDI-MSI is the chemical characterization and molecular mapping of microorganisms. Knowing such information is critical to gain high-value information regarding the function and properties of analytes. In the case of microbiology, MALDI-MSI lateral resolution might still be insufficient for bacterial single-cell characterization. However, such methodology could be suitable for larger organisms like parasites (e.g., *Schistosoma*) or diseases vectors (e.g., mosquitoes). To date, two publications related the molecular characterization and visualization of Schistosoma mansoni by MALDI-MSI [98,99]. It is worth noting that biomolecules such as lipids play a valuable role in the host recognition, immune response modulation, evasion, communication and development of S. mansoni [98]. Ferreira and colleagues characterized and differentiated the male and female of two *S. mansoni* strains from Brazil based on whole worm analysis. Clear differences between spectra were observed for the two strains, where most of the peaks were identified as lipids species, including triacylglycerols and phosphatidylcholines as the major classes. Additionally, the lipid composition seemed to vary according to the sex and the type of strain of the parasite. Along the same line, Kadesch and colleagues applied MALDI-MSI to characterize *S. mansoni* tegument surface-associated lipids [99] (Figure 5). The lipid composition was completely different from the inner and surface tissue. For example, higher abundances of sphingomyelins, phosphatidylserines, phosphatidylethanolamines and lysophosphatidylcholines were observed at the surface of the worms, while phosphatidylcholines and phosphatidylethanolamines were found to be more abundant inside the worms. In a close register, as the pathophysiology of *Plasmodium* infection relies on the use of a phospholipids host and the alteration of lipids content, a comprehensive characterization of the phospholipids topography of *Anopheles* mosquitoes is required [100]. Khali and colleagues also used MALDI-MSI to investigate the phospholipid composition of the *Plasmodium* vector *Anopheles stephensi* in mosquitoes. While phosphatidylcholines, phosphatidic acid and phosphatidylethanolamines were found to be abundant in the whole *Anopheles* body, sphingolipids and ether phospholipids, including ceramide-phosphatidylethanolamines, seemed to be characteristic in the head and antennal lobe.

Other authors introduced studies underlining that knowing organisms’ lipids composition and related localization could help in gaining greater knowledge of its life cycle and molecular mechanisms, as well as provide insights for the development of new drugs.

### 3.4. Drug Distribution and Effect

While developing and testing new drug molecules, researchers need to not only understand how they could be distributed or accumulated in pathogens or tissues, but also study the metabolization of the drug by the pathogen. In this sense, several research works were performed by MALDI-MSI applied to bacteria and parasites [70,102,103]. Morawietz and colleagues analyzed the spatial distribution of triclabendazole in the trematode *Fasciola hepatica* in order to identify the uptake route and tissue tropism of the drug [102].

**Figure 6:**
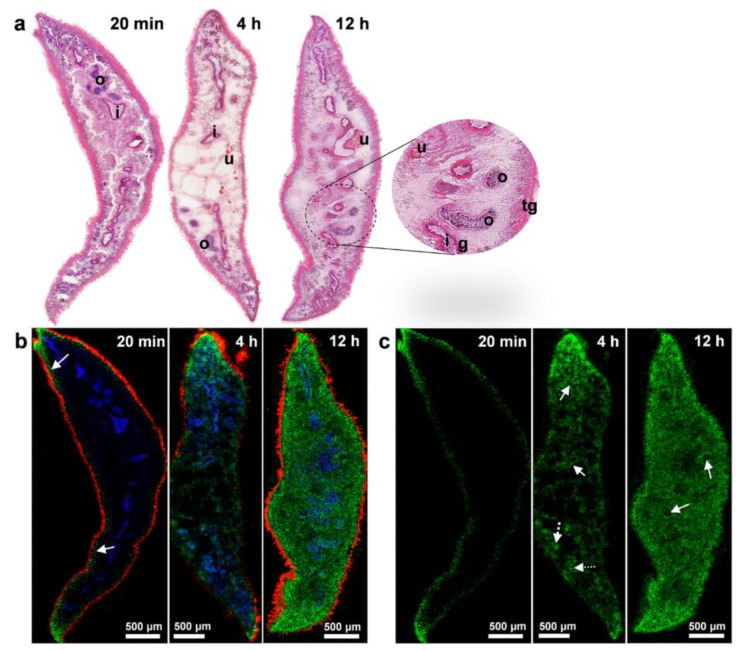
Kinetic of triclabendazole (TCBZ) uptake and distribution in *Fasciola hepatica*. Figure was reproduced from Morawietz and colleagues’ work [102].

After exposure to the drug, triclabendazole molecules were observed in the tegumental and sub-tegumental regions (20 min), and then further into tissues (4 h), until they formed a uniform distribution (12 h) in the parasite section, with the exception of eggs, where it was triclabendazole negative (Figure 6). As established in previous observations, the authors concluded that the triclabendazole uptake route was tegumental. Interestingly, different observations were made in their previous study focusing on imatinib [103]. Similarly, Phelan and colleagues analyzed the effect of the azithromycin antibiotic on *P. aeruginosa* colony biofilms metabolites production. While azithromycin is supposed to inhibit exchange of the molecules implied in *P. aeruginosa* quorum sensing, here, specialized metabolites production, i.e., quinolones 2-heptyl-4-quinolone, seems to increase following the azithromycin concentration gradient in susceptible strains [70].

Thus, by using MALDI-MSI to either track down drug molecules to understand their path and accumulation through the tissue, or their impact on the molecular machinery, such technology could be a powerful ally when it comes to testing new drug molecules.

### 3.5. Biomarker Identification and Diagnostics

As described in the previous section, MSI is mainly applied in life sciences as a mean to discover specific tumour or cancer biomarkers directly on tissue for diagnostics purposes [114]. Regarding the microbiology field, few studies were carried out to identify specific microbial biomarkers with a diagnosis aim. Among those publications, the majority focus on viruses [108,109,110,115]. In the book section “Application of mass spectrometry imaging in virus research”, Bertzbach and colleagues extensively explored the main applications of MSI in virology, with MALDI as the most widely used ionization source in the field [115]. Importantly, they underlined that MSI-based investigation of viruses involved in cancer accelerate identification of biomarkers. Nevertheless, among described applications, most references do not look directly at the virus itself and prefer to investigate pharmacological compounds in tissue sections. In contrast, Schwamborn and colleagues worked directly on the Pap smear for human papilloma virus, or HPV, for MALDI-MSI-based diagnostics [110]. By comparing positive and negative Pap smears, they identified five biomarkers and built two machine learning classifiers, i.e., a support vector machine and genetic algorithms, with an overall sensitivity and specificity of 88.9% and 71–78%, respectively. Their pioneering study highlighted the possibility of using MALDI-MSI to screen pathogenic microorganisms in a rapid and unbiased manner.

**Figure 7:**
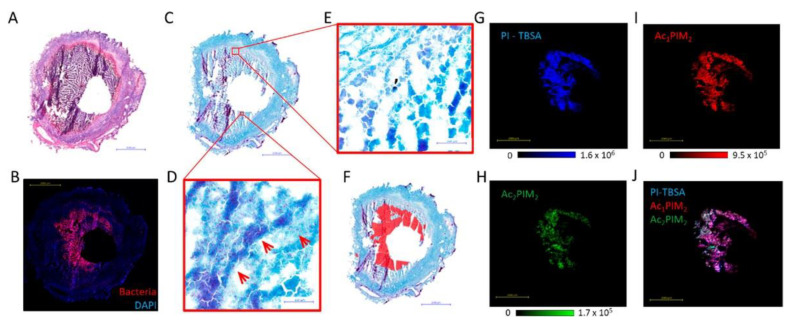
MALDI-MSI of phosphatidylinositol mannosides distributions within rabbit caseous-granuloma sections, matching *Mycobacterium* grouping. Figure was reproduced from Blanc and colleagues’ work [88].

While MALDI-MSI biomarkers discovery is mainly applied in virology, another study involving the visualization of mycobacterial biomarkers was performed [88]. Based on *Mycobacterium* spp. lipids fingerprint, described by Larrouy-Mamus and colleagues, phosphatidylinositol mannosides were investigated, as they were reported abundant within *M. tuberculosis* strains [116]. The distribution of phosphatidylinositol mannosides species, including Ac_1_PIM_2_ and Ac_2_PIM_2_, and phosphatidylinositol shared the same shape around the granuloma cavity, matching *Mycobacterium* distribution located by antibody-labelling (Figure 7). Therefore, such lipid species could be informative biomarkers while visualizing mycobacteria by MALDI-MSI. Interestingly, in the same study, the authors used the phosphatidylinositol biomarker to simultaneously image the distribution of both the *Mycobacterium* and anti-tuberculosis rifampicin drugs. In the case of rifampicin, the drug was observed throughout the tissue, including in the bacteria-dense area, underlining that the drug could penetrate and accumulate within bacteria-caseums.

Appealingly, the biomarkers approach described in this section could be potentially applicable for the visualization of other pathogens.

## 4. How Could It Be Applied to Overcome Current Limitations?

As stated above, current MALDI-TOF MS devices implemented in nearly all routine laboratories are not flawless. An area of uncertainty exists when it comes to analyzing closely related species and mixtures of microorganisms from complex biological samples, and understanding biological pathways hidden behind proteins peaks. Through the applications listed previously, MALDI-MSI might be a pertinent tool to overcome those limitations.

Regarding the identification of the closely related microbial species issue, MSI take up this challenge without any protein extraction steps. As a first hint, in the study of de Bruijn and colleagues, MALDI imaging was used to establish the metabolic fingerprints of several species and strains of *Lysobacter* directly on colonies. Different metabolic profiles were obtained for the different species, which matched gene clusters identified within the same work [61]. Obviously, further research is required for pathogens belonging to complex groups, such as *M. tuberculosis*, *M. abscessus* or *E. cloacae*, which are currently not distinguishable with the present MALDI-TOF MS. Nowadays, MALDI-TOF MS microbial experiments are based on the analysis of the 2–20kDa range, targeting mainly proteins and, more precisely, ribosomal proteins. The latest commercial devices released, when combined with artificial intelligence, enable the analysis of lipids, making it possible to discriminate species belonging to the Enterobacterales, i.e., *Shigella* spp. and *E. coli* [117]. However, the low-resolution associated with the linear TOF analyzers routinely implemented (approximately 5000) and the related mass accuracy might limit the discovery of biomarkers to delimitate the close frontier between two genetically close species. The majority of MALDI-MSI introduced studies worked with a hybrid (e.g., Q-Orbitrap) mass spectrometer (Table 1), meaning the instrument combines at least two mass analyzers of different types. In such configurations, the first analyzer (e.g., Quadrupole) could act as a mass filter and the second one (e.g., Orbitrap) could separate ions. The fact is that by using an orbitrap analyzer type, the work could be done under high resolution up to 500,000, i.e., providing the highest mass accuracy [118]. Therefore, such an analyzer enables the detection of peaks, which would be potentially merged while observed with a lower resolution analyzer, such as a TOF tube. Additionally, high resolution and the possibility to isolate upstream a parent ion and to subsequently perform one or several fragmentation experiments (e.g., *de novo* peptide sequencing by MS/MS), make it possible to identify and characterize biomarkers that could be linked directly to well-known biological mechanisms (e.g., antimicrobial resistances) [18]. Therefore, by using high resolution devices for MSI, it is possible to imagine screening agar plates quickly and accurately and then visualizing the identity of each colony under high throughput settings.

Whereas MALDI-TOF MS is mainly used on culture-grown colonies, direct analysis of biological samples, such as blood, requires the use of an extraction protocol before analysis. Nevertheless, for stools or other rich samples, the direct analysis of the microbial composition remains challenging. Experimental seeded stool with *Dientamoeba fragilis* was investigated by MALDI-TOF MS [119]. The authors reported that fecal material could interfere in the detection of specific *D. fragilis* proteins. Under MALDI-MSI imaging visualization for identification of macroscopic (e.g., parasites eggs) and microscopic (e.g., bacteria clusters) elements directly on humans or animals, stool smear should be considered. Indeed, the strong craze for artificial intelligence in recent years will allow the co-development of powerful diagnostics tools. While deep learning could recognize objects—in our case, parasites eggs—in digital photographs (e.g., several random pictures of a single stool smear), high-resolution MALDI-MSI could target microorganisms more precisely by the presence of certain lipids biomarkers, as in the study of Blanc and colleagues [88,120]. In comparisons of organs cross-section imaging, there is no need to screen the whole smear; a few plots of several pixels could be adequate to identify a multiplex of specific and defined microscopic pathogen biomarkers. Indeed, the average concentration of enteric pathogens in feces is high (e.g., 10^6^ CFU per ml for *Campylobacter* spp. infections), making each plot of pixels well-furnished for analysis. In the end, even if certain macroscopic elements end up with a restricted or suspicious identification, an additional confirmation could be done by MS. For example, as it is impossible to distinguish *Taenia saginata* from *Taenia solium* due to the identical morphology of their eggs under a microscope, a MALDI approach could possibly achieve such differentiation [17]. In addition to microorganisms identification, it might be worth it to also extend such logic to the detection of toxins. As an example, mycotoxins produced by molds, such as *Aspergillus fumigatus*, are frequently identified in food or beverage, making them a potential threat to human and animal health [121]. By using other laser-induced ionization methods, direct identification of metabolites produced by *A. fumigatus* on an infected spelt was achieved [121]. Therefore, while combined with artificial intelligence, MALDI-MSI could enable the elimination of the initial culture step from complex biological samples, as well as give the opportunity to analyze multiple organisms and their related important metabolites.

## 5. Outlooks and Future Challenges for MALDI-Imaging in Microbiology

MALDI-MSI is still in its infancy, and there are still challenges to address before applying it in routine microbiology or to exploring new microscopical aspects. In the following section, specific concerns and prospects for the future of MALDI-MSI in microbiology will be discussed.

### 5.1. Pragmatic Aspects

The majority of routine laboratories are currently working with mass spectrometers, either from Bruker Daltonics (Bremen, Germany) with the MALDI Biotyper, or from BioMérieux (Marcy-l’Etoile, France) with the VITEK MS and, more recently, the VITEK MS PRIME. In theory, if the appropriate imaging acquisition program is installed to pilot the laser and save the data, it might be possible to perform MSI on these devices. The analysis range of such apparatuses fluctuates from 2 to 20 kDa, which is suitable for the identification of microorganisms based on their protein fingerprints. However, TOF analyzers display relatively poor resolution and mass accuracy for low masses when mass resolution increases as *m*/*z* increases [122]. Hence, certain small metabolites could not be accurately visualized and identified because peptide fragmentation is not possible with these mass spectrometers. However, a TOF analyzer would be suitable for bigger molecules like proteins above 6–8 kDa, which is beyond the mass of range of an orbitrap analyzer. Publications investigated through this review (Table 1) use mass spectrometers equipped with an orbitrap analyzer. In contrast to the TOF analyzer, the orbitrap mass resolution decreases as *m/z* increases [123]. Hence, it has a very high mass resolution for low-weight compounds [122]. Nevertheless, despite the wide spectrum of commercial products offering different specifications (e.g., Orbitrap Exploris™ 120–240-480 or IQ-X, Fusion, Eclipse, Ascend tribrid MS from ThermoFisher Scientific), orbitrap mass spectrometers require the addition of a third-party (AP)-MALDI source, and they are pricier in comparison to already implemented MALDI-TOF devices in routine laboratories. Nevertheless, it is possible that, like sequencing, the price of orbitrap mass spectrometers will decrease with time and the willingness of manufacturers to expand to new markets.

### 5.2. The Importance of Standardizing Sample Preparation

Sample preparation is an essential step in MSI, as proper handling can save both distribution, i.e., prevent delocalization of analytes, and the abundance of biomolecules, as well as guarantee an optimized spatial resolution, a high sensitivity, better annotation and identification of molecules [122,124]. Nevertheless, to our knowledge, few studies focused on the optimization of MALDI samples preparation for the imaging of microorganisms [59,71,97]. In the introduced MALDI-MSI microbial studies, several parameters, such as samples fixation, matrix spraying and dehydration, were disparate.

One contrast between different studies is the choice of the substrate where the parasite sections or microbial colonies will be grown or fixed. Currently, stainless steel target plate and Indium-tin-oxide (ITO)-coated glass slides are used for MALDI-MSI analysis of microorganisms [74,95,97]. However, they do not have the same conductivity, which might have an impact later on the ionization/transmission of certain molecules. Subsequently, bacteria or fungi analysis require a growing step on agar. Three different methods are used in MALDI-MSI: (1) pouring agar and inoculating directly on the target, (2) embedding the target in agar prior to inoculation and incubation and (3) culturing the microbe in a petri dish, excising the region of interest and then transferring it to the target [59,60]. While agar could be avoided by cultivating samples directly on conductive silicon wafers or imprinting colonies with filter membrane, those were not described in the introduced studies [125,126].

One of the factor to guarantee an optimized spatial resolution is the size of the matrix crystals and its homogeneity[59,127]. In comparison to tissue sections or microbial protein extractions directly spotted on a target plate, applying the matrix directly to microbial colonies on agar might be tricky. 2,5-dihydroxy benzoic acid (DHB) and α-cyano- 4-hydroxycinnamic acid (CHCA), alone or mixed, are mainly employed for microbial studies. Interestingly, the matrix is sometimes dissolved in different solvents, such as ethanol, acetonitrile or methanol, and in different volume ratios and quantities [71,95]. The choice of matrix and solvent could have an important impact on the extraction and visualization of analytes, such as lipids [128]. Furthermore, different techniques exist for matrix applications. The first one is the use of a stainless steel sieve to dry coat and saturate microbial colonies [59]. Such an approach is low cost and efficient for a spatial resolution of 100 µm. However, the formation of matrix aggregates and uncovered spots suggest that sieve methods might be unreliable when a higher resolution needs to be reached [97]. In this sense, other methods, such as pneumatic sprayers and sublimation, were tested to increase the uniformity of the matrix coating. Automatic sprayers, such as ImagePrep, Bruker or SMALDIPrep, TransMIT GmbH, M3+-sprayer, HTX imaging or Suncollect, Sunchrom, allowed a better control of matrix application parameters, i.e., number of layers, distance or flow rate, by automatically spraying the sample in an enclosed chamber. Nevertheless, parameters change from one study to another [71,95]. The solid to vapor-phase transition, also known as sublimation, is also a well-known method to create a thin and homogenous layer of small matrix crystals (<1 µm) on samples [129]. Like the automatic sprayers, there are automated commercial solutions for a controlled sublimation (e.g., HTX Sublimator, HTX imaging). Such a method is commonly used for high spatial resolution MALDI-MSI analysis. An additional step, recrystallization, could also be applied after sublimation or matrix application. The target plate is placed in a pre-heated chamber with a paper soaked with solution, resulting in vapor for the recrystallization process [130]. Holzlechner and colleagues applied the matrix through a sublimation/recrystallization process for visualising fungal metabolites directly during myco-parasitic interactions by MALDI-MSI [74]. Interestingly, while sublimation is supposed to yield small matrix crystals and homogenous layers, pneumatic matrix spraying was reported as the technique of choice to reach down 1.4 µm lateral resolution [106]. In another context, under certain settings it is possible to omit the matrix application for analysis of bacterial colonies. Indeed, while most MALDI ionization sources are based on UV, the utilization of IR-lasers does not require the use of a matrix, opening up the possibility to explore native microbial systems, as well as biofilms on abiotic surfaces, such as catheters [81].

One of the downsides of imaging microorganisms grown on agar medium is the requirement to operate under high dehydration [38]. The dehydration step ensures that the source, mass analyser and detector of the mass spectrometer reach the required vacuum pressure for analysis [59]. While most studies perform dehydration at 37 °C, the incubation time could range from 20 min to 12 h, and, in some cases, drying was performed under pressure (e.g., 150 mbar) [68,70,71,84,97]. Nevertheless, dehydration may physically damage the sample by flacking it, i.e., fissures, air bubbles or detachment of the agar from the target plate [59]. In addition to physical damage, the degradation of endogenous metabolites during drying could also occur [95]. In this sense, Li and colleagues successfully accomplished eliminating the dehydration step for the imaging of biofilm cultured on agar media by using a one-step matrix application. After application of the matrix, the sample was dehydrated through the evaporation of the solvent and nitrogen gas flow. By avoiding heat-treatment, it was possible to enhance the imaging of metabolites localized in biofilm samples.

Overall, numerous ways exist to handle microbial samples for MALDI-MSI depending on the investigative objective. Further experimental design studies regarding important parameters, such as matrix spraying, type of support and the need to perform dehydration, should be enacted for microbial research. In this manner, standardized protocol could be established to evaluate the reproducibility and to compare results over different inter-laboratory studies.

### 5.3. Hardware and Bioinformatic Infrastructure: A Subject of Matter

One clear major limitation of applying MALDI-MSI to the microscopic world is directly linked to hardware acquisition parameters. Firstly, analysis of single-cell microorganisms is currently challenging due to the low lateral resolution of certain commercial devices. Lateral resolutions used in the publications described in Table 1 mainly range from 10 µm to 600 µm. Considering that the average size of bacteria, such as *E. coli*, is about 2 µm long and 0.5 µm diameter, efforts must be made to downscale lateral resolution. In this sense, certain microbial studies have already managed to reduce the lateral resolution up to 1.4 µm [106]. By coupling a specific focusing objective and an adjusted working distance, and optimizing matrix application, Kompauer and colleagues pointed out the possibility of detecting endogenic biomolecules ions in a 1.4 µm diameter spot. Whereas presented studies worked with reflection geometry, i.e., the laser focusing on the front, some adopt transmission geometry, i.e., the laser focusing on the back of the sample. By using such an approach, a lateral resolution of 600 nm was achieved within brain tissue [82]. Therefore, by adapting the laser geometry or optimizing the laser focusing, MALDI-MSI seems to be in a position to get closer to microscopic techniques. Additionally, the potential combination of single-cell imaging and single-cell sequencing was underlined as being able to give important insights regarding aquatic microbes and their environment [131]. Secondly, the limit of detection of microorganisms per pixel needs to be established. Indeed, pixel analysis relies on the average mass spectra of the different molecules present in the aforementioned pixel. Thus, if the microbial concentration is low, biomarkers might be missing. In the study of Blanc and colleagues, the limit of detection for mycobacterial lipid biomarkers was estimated around 5–10 bacteria per 50 µm^2^ pixel, as the lipid signal was not reproducibly detected when the concentration was fewer than 5–10 bacteria [88]. Authors also underlined that such a limit of detection may also rely on extraction efficiency. Finally, considering that MALDI technology is already widely implemented in most microbiology laboratories for routine identification, an important look must be given to the acquisition time. As mentioned in a previous section, MALDI-TOF MS takes around 15–20 min to identify 96 samples under routine settings. However, in the case of MALDI-MSI, image acquisition could be rather long depending on the size of the analyzed sample. It is estimated that for a 5 mm^2^ sample, a laser spot size diameter of 10 μm with adjacent ablation spots, and assuming 10 shots per pixel, could require an acquisition time of about 1.5 h [41]. In addition, the analysis time could dramatically increase under high resolution settings. Currently, mass spectrometers could acquire from 30 to 50 pixels per second regularly. Still, Bednařík and colleagues spotlighted the ability of MALDI-MSI to achieve acquisition rates up to 150 pixels per second due to a novel ion source’s design [132]. However, a high acquisition rate implies the lowest mass resolution, which limits identification ability. Therefore, MALDI imaging is always a complex balance between throughput and in-depth investigation of the local composition of biological tissues.

Along the same line, current devices used in routine microbiology laboratories are working under vacuum settings with a single ionization laser source. Interestingly, several publications described in Table 1 are using atmospheric pressure (AP) MALDI-MSI, i.e., the ionization takes place under atmospheric conditions [89,99,101,102,103,104,106]. The advantage to operating ionization at AP for MSI analysis is the elimination of the pumping time, the ease of sample preparation and the analysis of volatile molecules. Some manufacturers, such as MassTech© or TransMIT©, sell AP-MALDI sources that enable either efficiently switching from an LC/MS to an AP-MALDI-Orbitrap instrument and vice versa in few seconds (e.g., AP/MALDI (ng) UHR, MassTech©, Columbia, MD, USA) or transforming the mass spectrometers into a dedicated AP-MALDI Orbitrap instrument (AP-SMALDI5 AF, TransMIT©, Giessen, Germany). Hence, by skipping the pumping step, loading automatization of targets or glass slides could be achievable, resulting in a briefer analysis turnaround time and finer throughput. Additionally, AP might be the solution to avoid the problematic step of dehydration, which is imposed by the vacuum conditions of classical MALDI. Interestingly, certain AP-MALDI manufacturer source, such as MassTech©, offer portable, field-deployable and compact (weight: 16 kg) ion trap mass spectrometers with an AP interface (MT explorer 30, MassTech©) with the possibility to switch different exchangeable ionization sources, such as AP-MALDI or nanoESI. Such a compact device gives the opportunity to directly perform laboratory-level analysis on-site, which could be an asset when establishing temporary laboratories for humanitarian missions or for low-income laboratories. Overall, there is still a need to find the best trade-off between the different hardware acquisition parameters with a view to increase the throughput of MALDI-MSI so that it could be made available within a routine workflow one day.

Considering the ongoing digitalization and the key role of MALDI MS and other data-driven technologies in microbiology laboratories, a significant change in the analytical workflow in diagnostics laboratories is occurring [133,134]. Nonetheless, as alluded to in the first section, powered-omics methods require sufficient data storage infrastructure. Indeed, there is a need to find new ways to reduce data complexity, which is a burden when it comes to memory and computational power [135]. MSI datasets could be challenging to handle due to their large size and high degree of dimensionality. For example, a single 2D-MALDI-MSI dataset can reach around 1 GB, including 5000–50,000 spectra, while a 3D-MALDI-MSI dataset represents 10 to 100 2D-MALDI-MSI datasets, i.e., 100 GB per dataset [46]. In the case of raw high resolution, MSI file sizes can reach a few terabytes of spectral information [135]. Currently, several supervised and unsupervised methods exist for data compression (e.g., peak picking, segmentation, partial least squares classification or regression). Buchberger and colleagues described and explained most of these approaches extensively in their specialized review [43]. To briefly illustrate a data compression solution, peak picking algorithms are used to reduce data dimensionality and, therefore, its complexity by pulling out several peaks of interest. Nevertheless, even afterward, MSI datasets still have a high dimensionality. In this sense, Abdelmoula and colleagues developed an artificial neural network deep learning algorithm for unsupervised and peak learning of MSI data. By using such an approach, they managed to significantly reduce the spectral dimension from 730,403 to 61,343 *m*/*z* values of a 2D MALDI MSI dataset from human prostate cancer tissue samples [135]. Despite the application of such methods and the degree of dimensionality associated with biomedical MSI data, the identification of relevant features is growing demanding.

To wrap up, despite the difficulty ahead applying MALDI-MSI in a routine scenario in microbiology, MSI might be the next game-changer for all fields of microbiology, such as food surveillance and clinical, veterinary and environmental microbiology. Considering major challenges of the current century, such as antimicrobial resistances, MALDI imaging should be considered to prevent and tackle this silent pandemic by rapidly detecting molecules that are unseen while using current reference methods. Nonetheless, many questions remain open and further studies should specifically be conducted on sample preparation, hardware and software solutions. Finally, the dual combination of artificial intelligence and MALDI-MSI should be perceived as unavoidable for the utmost reliable, accurate and swift study of the microscopic world.

## Data Availability

Not applicable.
